# Analysis of epidemiological trends of and associated factors for tooth loss among 35- to 44-year-old adults in Guangdong, Southern China, 1995–2015: a population-based cross-sectional survey

**DOI:** 10.1186/s12903-023-02776-8

**Published:** 2023-02-05

**Authors:** Linxin Jiang, Jianbo Li, Zijing Yang, Xiaoyan Huang, Xiong Zhong, Yi Huang, Bincheng Liu, Linmei Wu, Shaohong Huang, Weihua Fan

**Affiliations:** Stomatological Hospital, Southern Medical University, Guangzhou, Guangdong People’s Republic of China

**Keywords:** Caries, Adults, Oral epidemiology, Missing teeth

## Abstract

**Background:**

Tooth loss is a known marker of oral and systemic health, but large-scale population-based and cross-sectional multi-year comparative studies on tooth loss have yet to be much studied in China. This study explores the changing trends in tooth loss status and the associated factors influencing the prevalence of tooth loss over the past two decades in Guangdong, Southern China.

**Methods:**

Data from three cross-sectional, representative oral epidemiological surveys in Guangdong Province were analyzed, including 400 in 1995, 720 in 2005, and 288 in 2015, for a total of 1408 participants. Sample selection is based on the National Census of China published by the National Bureau of Statistics. In this study, each year, the number of missing teeth (MT) and the prevalence of tooth loss (MT > 0) were calculated. Basic demographic information, socioeconomic status, caries and periodontal status, personal lifestyle factors, and dental health care behaviors were analyzed by multivariate logistic regression to estimate their associations with tooth loss. Statistical significance was evaluated with 2-sided tests with a significance level of *P* < 0.05.

**Results:**

This study found that the mean number of missing teeth and the prevalence of tooth loss among adults aged 35–44 years in Guangdong Province did not change significantly in the first decade (1995–2005) but decreased significantly in the second decade (2005–2015) (0.94 and 40.8% in 1995, 0.99 and 42.9% in 2005, and 0.63 and 33.3% in 2015, respectively). The mean number of MT by tooth position was highest for the first and second molars, and both were larger in the mandible than in the maxilla. In 1995, populations with low educational attainment and the presence of caries or periodontal pocket (periodontal probing depth ≥ 4 mm) were associated with a higher chance of MT > 0. In 2005, those with low educational attainment, the presence of caries, and 40–44 years old were associated with a higher chance of MT > 0. Moreover, in 2015, females, rural residents, and those with caries or periodontal pocket were associated with a higher chance of MT > 0.

**Conclusions:**

Although tooth retention has improved recently (2005–2015) and the preventive effect of education level on tooth loss has increased over time, efforts to prevent tooth loss in adults need to be strengthened. Particular attention should be given to preventive interventions for women, rural residents, and those suffering from caries or periodontal pocket.

## Background

Oral disease was one of the most common nonfatal health issues according to the Global Burden of Diseases hierarchy 2017 [1]. Tooth loss is one of the most prevalent and critical oral diseases worldwide [2]. The global prevalence and incidence of tooth loss have declined significantly in recent decades [3]. However, as of 2017, approximately 267 million people worldwide were suffering from complete edentulousness [4]. Tooth loss can have negative functional, aesthetic, and psychological consequences [5–7], reducing oral health-related quality of life [8]. It is also associated with anemia, cardiovascular disease, stroke, and end-stage renal disease [9–11]. The effects of tooth loss on other organs may result from factors associated with tooth loss leading to the release of inflammatory mediators. Tooth loss is therefore an essential indicator of oral and systemic health.

Several studies worldwide have monitored and analyzed tooth loss and found that many factors contribute to the occurrence of tooth loss (missing teeth > 0), such as socioeconomics factors (education level, income, and ethnicity) [12–14], lifestyle habits (smoking and sweet consumption) [15, 16], and oral disease presence (caries and periodontal pocket) [17]. The distribution of tooth loss and its influencing factors vary between countries and regions and are dependent on economic development, cultural, educational, and other factors. In China, although there has been a significant improvement in the status of tooth loss among adults compared with that a decade ago, the rate of missing teeth > 0 among adults aged 35–44 years was 32.3% in 2015 [18].

Three oral health surveys were conducted in Guangdong Province in 1995, 2005, and 2015 to investigate the status of tooth loss among adults aged 35–44 years. The rapid development of the social economy prompted many changes over these two decades. According to the Seventh National Census of China, the population aged 15–59 years in Guangdong Province increased by more than 10 million people compared to that in the Sixth National Census. In addition, the male-biased sex ratio, the proportion of the urban population and the proportion of people with nine years of education or more increased [19]. The classic diet is shifting, as the traditional dietary patterns of many young Chinese individuals are being influenced by Westernized diets, resulting in increased sugar intake [20]. Moreover, oral medical expenditure among Chinese adults was low, and basic medical insurance for oral health has not changed significantly in recent years [21]. The abovementioned factors may impact the population’s oral health in China. Although many studies on factors associated with tooth loss, both longitudinal and cross-sectional, have been conducted globally, only a few have been conducted in China. In China, studies on tooth loss were mainly epidemiological reports and analyses of factors in specific aspects (e.g., socioeconomic factors, particular diseases). Most of these samples were old adults. There has been no multi-year comparative analysis of epidemiological changes and associated factors on tooth loss in adults (35- to 44-year-olds) based on a large-scale population in China. This study was carried out to analyze the trend in tooth loss status and associated factors influencing the prevalence of tooth loss over the past two decades in Guangdong, Southern China.

A longitudinal study design can accurately observe the changes in trends and factors influencing tooth loss in the same sample and allows predictions, but it is costly and time-consuming. Moreover, there is a risk of loss to follow-up. By contrast, cross-sectional studies can still provide a good understanding of disease prevalence and its distribution characteristics at the time. Thus, this study analyzed data from three cross-sectional and representative oral epidemiological sample surveys conducted in 1995, 2005, and 2015 to explore the changing trends in tooth loss status and the associated factors influencing the prevalence of tooth loss over the past two decades in Guangdong, Southern China. Ex post stratified weights and multivariate logistic regression analysis were used to minimize cohort effects.

## Methods

### Source of data

Three epidemiological oral health surveys were conducted among adults aged 35–44 years in Guangdong Province in 1995, 2005, and 2015; the surveys were cross-sectional and representative. This study analyzed the data collected from the abovementioned surveys.

### Participants

The inclusion criteria were as follows: 35–44 years of age, residents of Guangdong Province (who had lived there for at least six months prior to the survey month), voluntarily participated, and signed informed consent.

The exclusion criteria were combined with severe systemic diseases (such as cardiovascular, digestive, respiratory, hematologic, and neurologic diseases) that would not cooperate with dental examination or questionnaire.

### Sample design

**Fig. 1:**
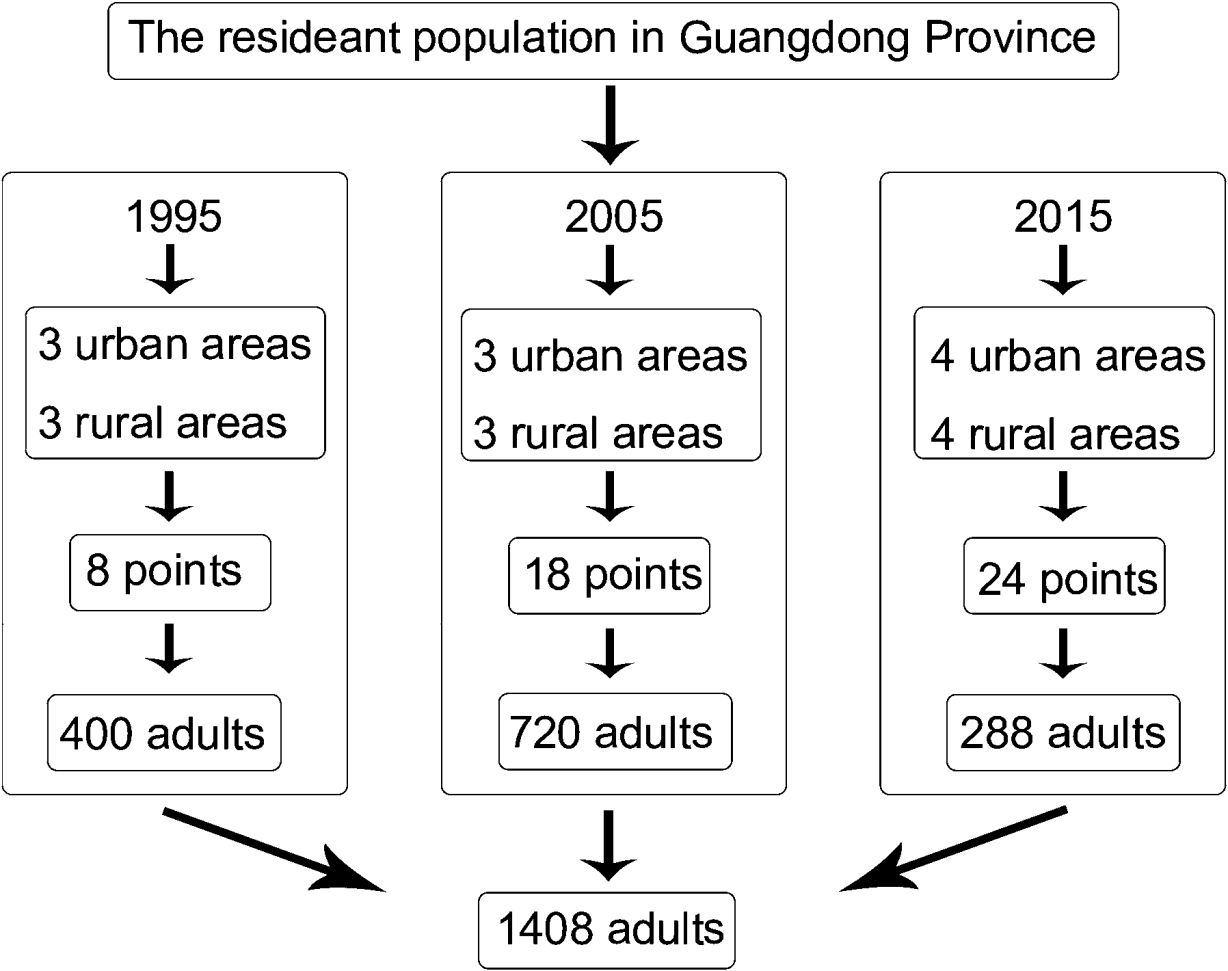
The sampling process

This study used stratified, multistage, cluster, and random sampling techniques to obtain samples from a representative population in Guangdong Province, China. In 1995, 3 urban areas and 3 rural areas in Guangdong Province were randomly selected; 4 streets/towns were selected in urban or rural area, for a total of 8 study sample points. A total of 50 adults were sampled per point, for a total of 400 adults. In 2005, 3 urban areas and 3 rural areas in Guangdong Province were randomly selected; 3 streets/towns were selected in all area, for a total of 18 study sample points. A total of 40 adults were sampled per point, for a total of 720 adults. In 2015, 4 urban areas and 4 rural areas in Guangdong Province were randomly selected; 3 streets/towns were selected in all area, for a total of 24 study sample points. A total of 12 adults were sampled per point, for a total of 288 adults. Overall, 400 adults were included in the final sample in 1995, 720 in 2005, and 288 in 2015, for a total of 1408 individuals. And the ratios of males to females and urban areas to rural areas were both 1:1 (Fig. 1).

### Dental examination design

Dental examinations were carried out by 3 examiners and recorded by 3 assistants for each survey year. All of the examiners were experienced dentists who had practiced clinical work for more than 3 years. Before the surveys, 3 examiners were provided training and initial calibration by a standard examiner. The training content was divided into two parts and completed in two central pieces of training: theoretical study and clinical practice of caries examination standards. The kappa values of the three examiners to the standard examiner for caries examinations were > 0.8 and for periodontal examinations were > 0.6.

The standard examination equipment included an examination light, a dental mirror, and a community periodontal index (CPI) probe. All visible teeth except the third molars were checked in the following order: #17 to #27, then #37 through #47. The diagnostic criteria applied in this study referred to the World Health Organization (WHO) oral health survey basic methods (3^rd^, 4^th^, and 5^th^ edition) and are as follows:

- Crown caries/root caries: crown caries was defined as an obvious cavity, underenamel destruction, or a softened lesion at the crown that was detected on the base or wall. Root caries was defined as CPI probe detection of root cementum destruction or lesions with a soft or leathery feel.
- Periodontal pocket: periodontal pocket was defined as periodontal probing depth of ≥ 4 mm.
- Tooth loss status: missing teeth due to caries or other causes were defined as MT. (In this study, MT > 0 was defined as the prevalence of tooth loss, and the outcome variable was treated as binary variable in this study (“MT = 0” and “MT ≥ 1”)).

### Questionnaire design

Trained interviewers conducted the in-person, one-on-one questionnaire. A centralized and standardized training was conducted before questionnaire administration, and consistency among interviewers after training was > 95%. The training content includes clarifying the purpose and meaning of the questionnaire, understanding the principles and methods of questionnaire design, unifying the questionnaire indicators and filling requirements, and standardizing the procedures and methods of questioning. The questionnaire answers for the three surveys were not the same. Thus, in this study, common variables for all three questionnaires were identified, and the final inclusion variables were grouped into four categories, as follows: (1) basic demographic information, including name, sex, and age; (2) socioeconomic status, including registered permanent residence type, education level, and annual household income; (3) personal lifestyle factors, including oral hygiene practices, sweets consumption, smoking, and alcohol consumption; and (4) dental health care behaviors, including the time since last dental visit, the reason for the last dental visit (within a year), and the payment mode of the dental visit (within a year).

Age was divided into two groups: 35–39 years old and 40–44 years old. Registered permanent residence type could be classified into two categories: urban area and rural area. Education level was divided into two categories by the number of years at school, with the cutoff being the median years per survey: low educational attainment (≤ 9 years, graduation from junior high school or less) and high educational attainment (> 9 years, more than graduation from junior high school). Annual household income was categorized into 3 levels by quartiles per survey: low income (< quartile 1), medium income (quartile 1 to quartile 3), and high income (> quartile 3). Tooth brushing was categorized by frequency as ≤ once per day or ≥ twice per day. Dental flossing was categorized by frequency as yes or no. Toothpaste was categorized into fluoride toothpaste or nonfluoride toothpaste. Sweets consumption, which included the consumption of sweet snacks and sweet beverages, was classified by frequency into three levels: rarely, sometimes (< twice per day), and often (≥ twice per day). Smoking and alcohol consumption were classified as yes or no for each. The time since last dental visit was categorized by frequency as < one year or ≥ one year. The reason for the last dental visit (within a year) was categorized as treatment, consultation, or prevention. Dental visit payment mode (within a year) was classified as entirely out of pocket and nonfully out of pocket.

### Statistical analysis

Due to the different distribution of the total population among the regions, post hoc stratification was required to ensure the sample population was representative of that region’s total population and to adjust for deviations in the sample and overall distribution of important indicators caused by sampling. This method assigns ex post stratified weights to each sample, with the sample distributions of indicators by weight consistent with the overall distribution. The ex post stratified weighted overall population was the resident population of each city in Guangdong Province in 2010, with information obtained from China’s National Bureau of Statistics. Populations were stratified by sex (male and female) at each study sample point to improve the accuracy of the weights, and the stratification weights were calculated as follows: W = the proportion of males and females in the overall population of each city by sex/the proportion of males and females in the sample population of the corresponding measure. Data from this study were analyzed after being weighted and standardized using the above weightings.

Chi-square tests and Wilcoxon rank-sum tests were used to compare differences in the rates of MT > 0 and the mean numbers of MT among subgroups. The factors associated to tooth loss were analyzed by multivariate logistic regression analysis, including a summary section and a section stratified by the year of surveys. For the analysis of the summary section, six interaction terms generated by the year and the independent variable was added to the regression analysis to detect the possible interactions with the survey period (years × age, years × sex, years × residence, years × education level, years × caries, years × periodontal pocket). Odds ratios (*OR*s) and 95% confidence intervals (95% *CI*s) were estimated. The α level for statistical significance was set to 0.05. All statistical analyses were performed using IBM SPSS Statistics version 25.0.

## Results

### Tooth loss status

#### Mean number of MT and prevalence of tooth loss

**Fig. 2:**
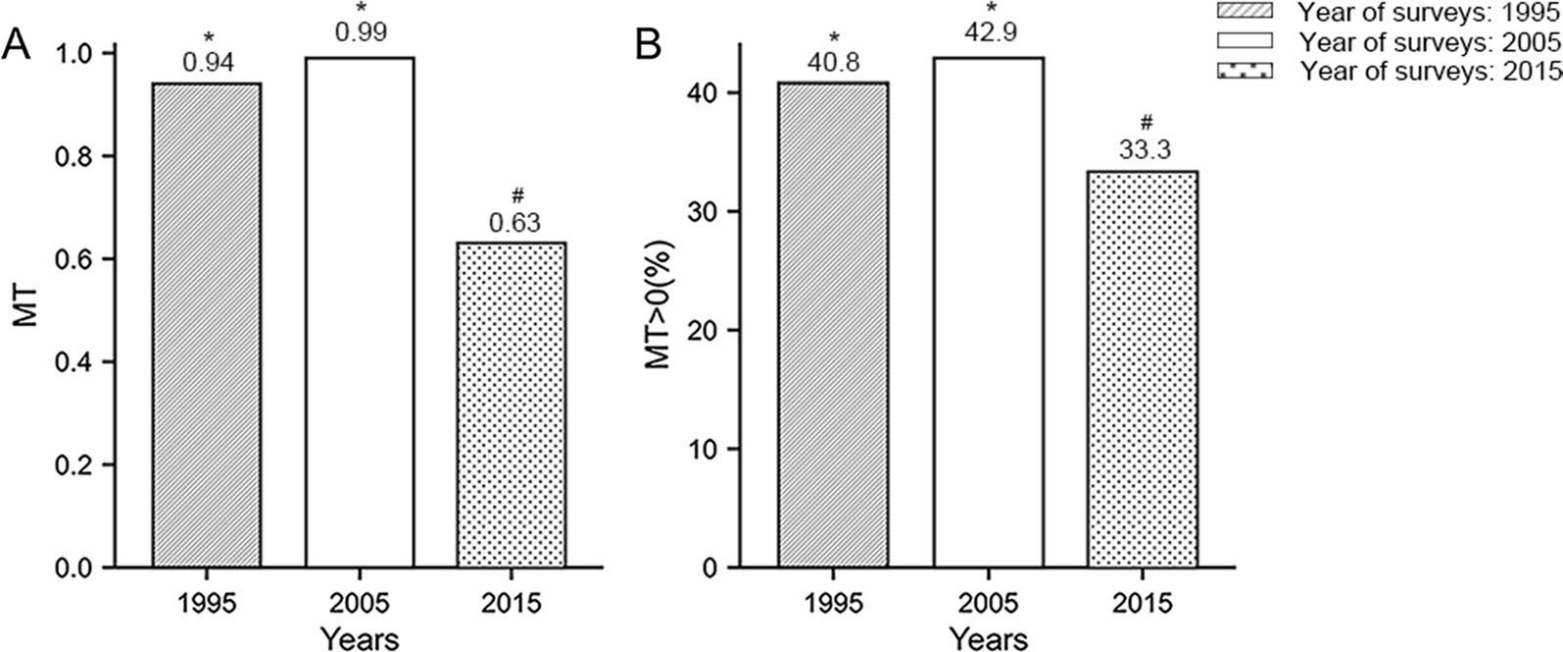
The changing trends of tooth loss. **A** Mean number of MT in different years of surveys; **B** Prevalence of tooth loss (MT > 0) in different years of surveys. Different characters (*, #) indicate significant differences between survey years (*p* < 0.05)

The overall distributions of the mean number of MT (*H* = 7.73, *P* = 0.021) and the rate of MT > 0 (*χ*^*2*^ = 7.89, *P* = 0.019) from 1995 to 2015 showed statistically significant differences. The changes from 1995 to 2005 were nonsignificant (*P* > 0.05); the mean number of MT and the rate of MT > 0 slightly increased from 0.94 and 40.8% to 0.99 and 42.9%, respectively. Moreover, there was a significant decrease from 2005 to 2015 (*P* < 0.05) from 0.99 and 42.9% to 0.63 and 33.3%, respectively (Fig. 2).

The overall characteristics of the survey participants in 1995, 2005, and 2015 are displayed in Table 1. In 1995, the sex differences in the mean number of MT and the rate of MT > 0 were not statistically significant, but the mean number of MT was higher in rural than in urban areas (*Z* = -2.35, *P* = 0.02). In 2005, the regional differences in the mean number of MT and the rate of MT > 0 were not statistically significant, but the mean number of MT and the rate of MT > 0 were higher in females than in males (*Z* = -2.58, *P* = 0.01; *χ*^*2*^ = 5.15, *P* = 0.02). In 2015, the mean number of MT and the rate of MT > 0 were higher in rural than urban areas and in females than males, with statistically significant differences.

**Table 1:** Tooth loss status distribution of 35–44 years adults in Guangdong Province by residence areas, sex

Year of surveys	Variable		N	MT > 0		MT	
				N (%)	*P* value^a^	${\overline{\text{x}}} \pm {\text{s}}$	*P*-value^b^
Year of surveys: 1995	Total		400	163 (40.7)		0.94 ± 1.64	
	Residence	Urban	200	74 (37.0)	0.127	0.68 ± 1.14	0.019*
		Rural	200	89 (44.5)		1.21 ± 1.99	
	Sex	Male	203	74 (36.5)	0.076	0.85 ± 1.67	0.083
		Female	197	89 (45.2)		1.04 ± 1.62	
Year of surveys: 2005	Total		720	309 (42.9)		0.99 ± 1.75	
	Residence	Urban	361	152 (42.1)	0.659	0.97 ± 1.68	0.694
		Rural	359	157 (43.7)		1.00 ± 1.82	
	Sex	Male	359	139 (38.7)	0.023*	0.89 ± 1.77	0.010*
		Female	361	170 (47.1)		1.09 ± 1.74	
Year of surveys: 2015	Total		288	96 (33.3)		0.63 ± 1.13	
	Residence	Urban	144	38 (26.4)	0.009*	0.51 ± 1.03	0.023*
		Rural	144	59 (41.0)		0.74 ± 1.21	
	Sex	Male	158	42 (26.6)	0.005*	0.47 ± 0.99	0.003*
		Female	130	55 (42.3)		0.83 ± 1.25	

^a^Chi-squared test

^b^Wilcoxon rank-sum test

*Statistical significance *p* < 0.05

#### Mean number of MT at different tooth positions

**Fig. 3:**
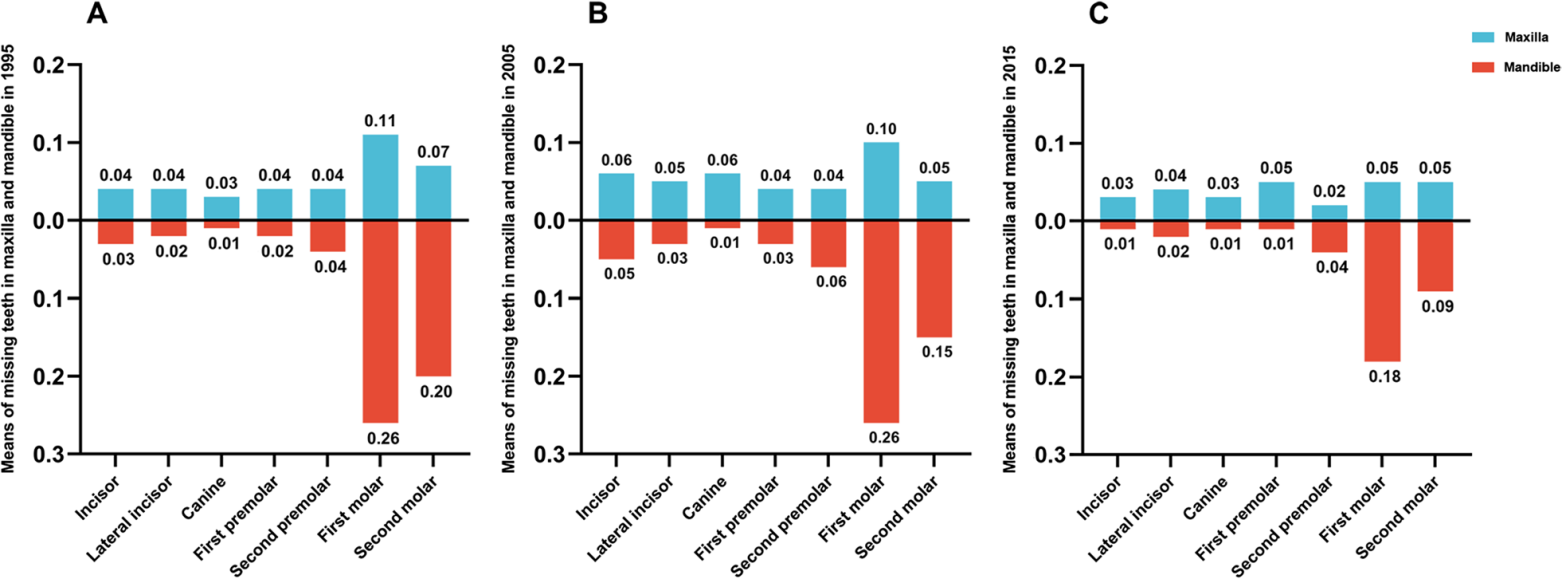
**A** The mean number of MT in maxilla and mandible in 1995; **B** The mean number of MT in maxilla and mandible in 2005; **C** The mean number of MT in maxilla and mandible in 2015

The mean numbers of MT at all tooth positions in the different survey years are shown in Fig. 3A–C.

Among all the tooth positions, the first molar was the most common position in 1995, 2005, and 2015, followed by the second molar, and both were higher in the mandible than in the maxilla. In contrast, the mean numbers of MT in the anterior and first premolar positions were higher in the maxilla than in the mandible.

#### Univariate analysis

The result of the univariate analysis is shown in Table 2 and is discussed considering four categories.

**Table 2:** Univariate analysis of factors associated to MT > 0 among 35–44 years adults in Guangdong Province

Variables	N (%)	MT > 0 (%)	*P*-value^a^
Age			
35–39 years old	720 (51.1)	252 (35.0)	< 0.001*
40–44 years old	688 (48.9)	317 (46.1)	
Year of surveys			
1995	400 (28.4)	163 (40.8)	0.019*
2005	720 (51.1)	309 (42.9)	
2015	288 (20.5)	96 (33.3)	
Sex			
Male	720 (51.1)	255 (35.4)	< 0.001*
Female	688 (48.9)	313 (45.6)	
Residence			
Urban	705 (50.0)	264 (37.4)	0.025*
Rural	704 (50.0)	305 (43.3)	
Education level			
Low educational attainment	800 (56.8)	363 (45.4)	< 0.001*
High educational attainment	608 (43.2)	205 (33.7)	
Annual household income			
Low income	457 (32.7)	196 (42.9)	0.404
Medium income	687 (49.2)	270 (39.3)	
High income	253 (18.1)	98 (38.7)	
Caries			
No	552 (39.2)	129 (23.4)	< 0.001*
Yes	856 (60.8)	439 (51.3)	
Periodontal pocket			
No	981 (69.7)	369 (37.6)	0.001*
Yes	427 (30.3)	199 (46.7)	
Tooth brushing			
≤ Once per day	656 (46.6)	273 (41.6)	0.403
≥ Twice per day	752 (53.4)	296 (39.4)	
Dental flossing			
No	1375 (97.8)	555 (40.3)	0.855
Yes	31 (2.2)	12 (38.7)	
Toothpaste			
Nonfluoride toothpaste	744 (53.2)	301 (40.5)	1.000
Fluoride toothpaste	656 (46.8)	265 (40.4)	
Sweet consumption			
Rarely	221 (16.9)	78 (35.3)	0.256
Sometimes	953 (72.8)	392 (41.1)	
Often	136 (10.3)	57 (41.9)	
Smoking consumption			
No	943 (67.0)	388 (41.1)	0.434
Yes	465 (33.0)	181 (38.9)	
Alcohol consumption			
No	801 (57.0)	336 (41.9)	0.161
Yes	604 (43.0)	231 (38.2)	
Time since last dental visit			
< one year	269 (29.7)	146 (54.3)	0.276
≥ one year	638 (70.3)	321 (50.3)	
The reason of the last dental visit (within a year)			
Treatment	249 (93.1)	137 (55.0)	0.223
Consultation	12 (4.5)	4 (33.3)	
Prevention	6 (2.4)	5 (71.4)	
The payment mode of the dental visit (within a year)			
Nonfully out of pocket	56 (20. 8)	27 (48.2)	0.290
Entirely out of pocket	212 (79.2)	119 (56.1)	

^a^Chi-squared test

*Statistical significance *p* < 0.05

#### Socioeconomic status

A total of 45.4% of adults with low educational attainment suffered from tooth loss; this proportion was 11.7% higher than that in those with high educational attainment (*P* < 0.001). The sample distribution of annual household income was 32.7% for low income, 49.2% for medium income, and 18.1% for high income. The prevalence of tooth loss decreased with increasing income level, but the difference was not statistically significant.

#### Caries and periodontal status

In total, 60.8% of adults suffered from caries, and 30.3% suffered from periodontal pocket. The prevalence of tooth loss was higher in those with caries than in those without (*P* < 0.001) and in those with periodontal pocket than in those without (*P* = 0.001).

#### Personal lifestyle factors

In total, 53.4% of adults brushed their teeth ≥ twice per day, 46.8% used fluoride toothpaste, and only 2.2% flossed. The above results suggest that the rates of tooth brushing and the use of fluoride toothpaste are acceptable, but the practice of dental flossing needs to be popularized. The prevalence of tooth loss was lower among those who brushed ≥ twice per day, used fluoride toothpaste and flossed than among those who brushed ≤ once per day, used nonfluoride toothpaste, and did not floss. Those who frequently consumed sweets had a higher prevalence of tooth loss than those who rarely or sometimes consumed sweets. The prevalence of tooth loss was 2.2% lower among smokers than nonsmokers and 3.7% lower among alcohol drinkers than among nondrinkers. None of these differences were statistically significant.

#### Dental health care behaviors

Most adults had not visited the dentist within a year since their last visit, with only 29.7% reporting a dental visit within the past year. Among those who had visited the dentist within a year, 93.1% visited to seed treatment, while only 6.9% visited for consultation or prevention purposes. Furthermore, 79.2% of adults had to pay for the cost of the dental visit out of pocket. None of these differences were statistically significant.

#### Regression analysis

Variables with a *P* value < 0.05 in the univariate analysis were included in the multivariate logistic regression models. Survey year was included in the model as continuous variable to explore year-to-year changes better.

The multivariate logistic regression analysis showed that aged 40–44 years, females and caries or periodontal pocket were significantly associated with a higher chance of MT > 0. High educational attainment was significantly associated with a lower chance of MT > 0 (Table 3). When analyzing the interaction between the included variables and survey year, years × education level and years × caries were significant and added as two interactive variables to the regression analysis. Respectively, the significant interactions between education level and years indicated that from 1995 to 2015, the effect of high education attainment on a lower chance of MT > 0 increased. And the significant interactions between caries and years indicated that from 1995 to 2015, the impact of caries on a higher chance of MT > 0 decreased.

**Table 3:** Multivariate logistic regression analysis of factors with MT > 0 among 35–44 years adults in Guangdong Province

Variables	MT > 0 (%)	OR (95%CI)
Years		
	–	1.03 (0.99–1.07)
Age		
35–39 years old^†^	35.0	–
40–44 years old	46.1	1.41 (1.12–1.77)
Sex		
Male^†^	35.4	–
Female	45.6	1.29 (1.02–1.63)
Residence		
Urban^†^	37.4	–
Rural	43.3	1.09 (0.86–1.39)
Education level		
Low educational attainment^†^	45.4	–
High educational attainment	33.7	0.52 (0.35–0.78)
Caries		
No^†^	23.4	–
Yes	51.3	7.04 (4.51–10.98)
Periodontal pocket		
No^†^	37.6	–
Yes	46.7	1.53 (1.19–1.96)
Education level × years		
	–	1.04 (1.01–1.07)
Caries × years		
	–	0.93 (0.89–0.96)

^†^Reference category

*OR* odds ratio, *CI* confidence interval

**Fig. 4:**
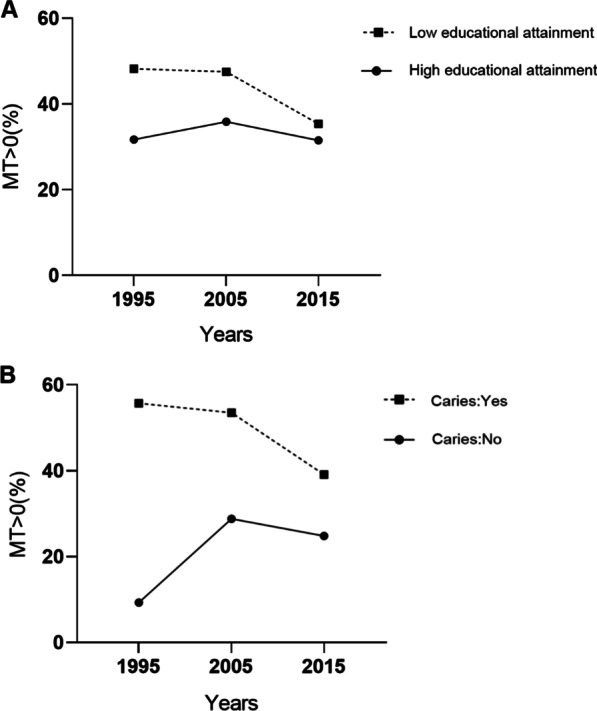
Trends in the prevalence of tooth loss (MT > 0). **A** Trends in education attainment in different years of surveys; **B** Trends in caries in different years of surveys

The above interaction results are consistent with the trends in Fig. 4. In Fig. 4A, there is a trend that the gap in the prevalence of tooth loss between the high and low educated population decreased. In Fig. 4B, there is a trend that the gap in the prevalence of tooth loss between the populations with and without caries decreased.

After stratification by survey year, the logistic regression subgroup analyses showed that those with low educational attainment and those suffering from caries or periodontal pocket had a higher chance of t MT > 0 in 1995. In 2005, those with low educational attainment, the presence of caries, and 40–44 years old were associated with a higher chance of MT > 0. In 2015, females, rural residents, and those with caries or periodontal pocket had a higher chance of t MT > 0 (Table 4).

**Table 4:** Multivariate logistic regression analysis of factors with MT > 0 among 35–44 years adults in Guangdong Province stratified by the year of surveys

Variables	1995			2005		2015	
	MT > 0 (%)		OR (95%CI)	MT > 0 (%)	OR (95%CI)	MT > 0 (%)	OR (95%CI)
Age							
35–39 years old^†^		39.8	–	34.4	–	29.9	–
40–44 years old	41.8		0.80 (0.50–1.29)	52.3	1.81 (1.32–2.48)	37.1	1.52 (0.90–2.56)
Sex							
Male^†^	36.5		–	38.7	–	26.6	–
Female	45.2		1.14 (0.71–1.83)	47.1	1.13 (0.82–1.56)	42.3	2.04 (1.21–3.46)
Residence							
Urban^†^	37.0		–	42.1	–	26.4	–
Rural	44.5		1.09 (0.67–1.80)	43.7	0.86 (0.62–1.20)	41.0	2.63 (1.43–4.84)
Education level							
Low educational attainment^†^	48.2		–	47.5	–	35.4	–
High educational attainment	31.7		0.43 (0.26–0.73)	35.9	0.67 (0.48–0.94)	31.5	1.62 (0.88–2.98)
Caries							
No^†^	9.3		–	28.8	–	24.8	–
Yes	55.7		13.76 (7.13–26.56)	53.5	2.63 (1.89–3.67)	39.1	2.07 (1.18–3.63)
Periodontal pocket							
No^†^	37.5		–	40.0	–	30.1	–
Yes	49.5		1.97 (1.16–3.34)	50.8	1.39 (0.96–1.98)	38.2	1.77 (1.04–3.02)

*OR* odds ratio, *CI* confidence interval

^†^Reference category

## Discussion

The main finding of the study was that the following factors influenced the prevalence of tooth loss among survey years: educational level, caries, and periodontal pocket in 1995; age, educational attainment, caries in 2005; and sex, residence, caries, and periodontal pocket in 2015. For the analysis of the interaction terms, years × education level and years × caries were significant. This means that while there are factors that persist over time, there are also inequality factors that change over time.

In this study, the mean number of MT and the prevalence of tooth loss in 1995 and 2005 were nonsignificant differences, but they significantly decreased from 2005 to 2015. The decline may be due to the increased investment in health services and the effectiveness of oral prevention strategies in China. The mean number of MT in 2015 was 2.3, and the prevalence of MT > 0 was 75.5% in adults aged 35–44 in China [18]. In the UK, the number of remaining teeth in adults increased from 21.9 in 1968 to 25.7 in 2009 [22]. In Germany, the prevalence of missing teeth > 0 decreased from 76.6% in 1997 to 58.8% in 2005 and to 56.8% in 2014 [23]. In addition, the mean number of MT in Brazil decreased from 13.5 in 2002–2003 to 7.4 in 2010 [24]. The studies summarized above show that the status of tooth loss among adults in Guangdong Province is relatively better than those in China overall and abroad and that there is a trend toward improvement. However, this may also be associated to the fact that residual roots and crowns were not considered missing teeth at the time of the surveys utilized in this study. Meanwhile, The mean number of MT among all tooth positions in the three surveys was highest for the first and second molar and was particularly significant in the mandible. This result is similar to domestic and international findings that emphasize the larger number of missing teeth at the molar position [18, 25, 26].

Differences in the prevalence of tooth loss by sex and residence were not statistically significant between 1995 and 2005. However, the chance of tooth loss was about 2.04 times higher in females than in males and about 2.63 times higher in rural areas than in urban areas in 2015, with similar results reported in other studies [18, 27, 28]. There were sex differences between two studies conducted in Iran and Vietnam [29, 30], and there were residency differences in a study in the Netherlands [31], whose results are contrary to our study. The differences in the sex and residency results may be associated to different countries’ cultures and economic development levels.

Education level is an essential indicator of inequality and this topic has been explored in several studies [32–34]. The chance of tooth loss in 1995 and 2005 were about 0.43 and 0.67 times lower, respectively, in adults with high educational attainment than in those with low educational attainment. In contrast, the difference in the prevalence of MT > 0 between education levels in 2015 was not statistically significant. The results in 1995 and 2005 suggest that higher educational attainment is beneficial in preventing tooth loss, consistent with the findings of other national studies [23, 35, 36]. For the results in 2015, this study inferred that the main reason was that the increase in education level over time reinforced its preventive effect on tooth loss. There are several reasons for this inference. First, multivariate logistic regression analysis showed a significant interaction between education level and years. That means high education attainment's preventing effect on tooth loss prevalence was enhanced over time. Second, the difference in the prevalence of tooth loss by educational level showed a significant tendency to decrease with the years. Thirdly, the distribution of the number of years of education in this study showed that the proportion of those with > 9 years of education was less than the proportion of those with ≤ 9 years of education in 1995 and 2005, but the two categories contained the same proportion in 2015. The abovementioned changes may be explained by the nine-year compulsory education policy, which has been implemented in China since 1986, suggesting that the average education level increased significantly and the inequality in the impact of education level on tooth loss decreased over the two-decade study period. It is believed that the education levels of 35- to 44-year-old adults in Guangdong Province will gradually increase, which may have a positive impact on the situation of tooth loss.

Caries is, along with tooth loss, the most prevalent and vital oral disease worldwide [2]. It is also the leading cause of tooth loss [2]. The presence of caries was the only associated factor in all three surveys. In exploring the interaction between caries and time, it was shown that the negative effect of caries on the prevalence of tooth loss over time significantly diminished. The chances of tooth loss among those with caries in the three surveys were about 13.76 times, 2.63 times, and 2.07 times higher than those among people without caries, with caries rates of 67.7%, 57.0%, and 60.7%, respectively. And the differences in the prevalence of tooth loss by caries showed a tendency to decrease with the year of the surveys. The above results showed a decreasing trend, suggesting that caries prevention strategies were effective. However, the caries rate in 2015 was higher than that in 2005, suggesting that the impact of caries on tooth loss cannot neglected, and prevention and treatment measures for caries should be strengthened. This is the same view as in a prospective population-based cohort study in Brazil: tooth loss is the result of the progression and accumulation of treatments requiring tooth decay, and it is possible to avoid tooth loss by investing in effective public policies dealing with prevention and treatment in the early stages of dental caries [37]. Besides, it is important to note the connection between caries and periodontal disease. The chances of tooth loss among those with periodontal pocket were 1.97 and 2.77 times higher than those without periodontal pocket in 1995 and 2015. The periodontal pocket rates were 26.7%, 27.4% and 42.4%, respectively, with a significant increase in 2015. A previous review emphasized the negative role of root caries in the long-term preservation of teeth in patients with periodontal disease, and another study analyzed the risk of root caries in patients with periodontitis [38, 39]. The results indicate an interactive effect between root caries and periodontitis. However, the relationship between crown caries and periodontal disease is still not clear and has been reported to be positive, negative, and unrelated [40–43]. In a recent article from China on the relationship between caries and periodontal disease, it was stated that among people aged 35–44 years, periodontitis had a significant association with mixed or root caries [44]. Therefore, the interaction between caries and periodontal disease needs to be recognized to prevent the adverse effect of tooth loss.

This study has several limitations. First, this study used data from three cross-sectional rather than continuous prospective data; thus, it cannot accurately predict the status of tooth loss in the next decade. Second, given the deviations in the samples and overall distributions of important indicators caused by the sampling method, this study assigned ex post stratified weights to each sample. Additionally, it explored the potential causal effects of confounding factors in each survey year. Nevertheless, there may still be bias in the results due to differences associated with time periods and economic development levels. Finally, the three surveys included different dental examination items and questionnaire contents, resulting in a reduction in the number of common variables and preventing comprehensive analysis of the prevalence of tooth loss from all perspectives.

## Conclusions

In summary, the epidemiological status of tooth loss among adults aged 35–44 years in Guangdong Province in 2015 significantly improved compared with those in 1995 and 2005. At the same time, there is a clear trend at the educational level that the preventive effect of education level on tooth loss has increased over time, and the inequality in the prevalence of tooth loss in terms of education level has gradually decreased. However, additional precautions for specific populations are necessarily based on factors associated with tooth loss. These populations include women, those living in rural areas, and those suffering from caries or periodontal pocket.

## Data Availability

The data that support the findings of this study are available on request from the corresponding author [Fan W]. The data are not publicly available due to them containing information that could compromise research participant privacy.

## Abbreviations

MT: Missing teeth
CPI: Community periodontal index
WHO: World Health Organization

## References

[1] Global, regional, and national incidence, prevalence, and years lived with disability for 354 diseases and injuries for 195 countries and territories, 1990–2017: a systematic analysis for the Global Burden of Disease Study 2017. Lancet. 2018;392(10159):1789–1858. 10.1016/S0140-6736(18)32279-7 PMC6227754 30496104

[2] Peres MA, Macpherson L, Weyant RJ (2019). Oral diseases: a global public health challenge. Lancet.

[3] Kassebaum NJ, Bernabé E, Dahiya M, Bhandari B, Murray CJ, Marcenes W (2014). Global burden of severe tooth loss: a systematic review and meta-analysis. J Dent Res.

[4] Bernabe E, Marcenes W, Hernandez CR (2020). Global, regional, and national levels and trends in burden of oral conditions from 1990 to 2017: a systematic analysis for the global burden of disease 2017 study. J Dent Res.

[5] Bousiou A, Konstantopoulou K, Polychronopoulou A, Halazonetis DJ, Schimmel M, Kossioni AE (2022). Sociomedical and oral factors affecting masticatory performance in an older population. Clin Oral Investig.

[6] Muhammad T, Srivastava S (2022). Tooth loss and associated self-rated health and psychological and subjective wellbeing among community-dwelling older adults: a cross-sectional study in India. BMC Public Health.

[7] Bulgarelli AF, Dos SC, Rech RS, Baumgarten A, Goulart BN (2021). Tooth loss condition and social discrimination in brazilian healthcare services. Int J Public Health.

[8] Tan H, Peres KG, Peres MA (2016). Retention of teeth and oral health-related quality of life. J Dent Res.

[9] Han K, Park JB (2018). Evaluation of the association between the number of natural teeth and anemia among Korean adults using nationally representative data. J Periodontol.

[10] Cheng F, Zhang M, Wang Q (2018). Tooth loss and risk of cardiovascular disease and stroke: a dose-response meta analysis of prospective cohort studies. PLoS ONE.

[11] Han K, Park JB (2021). Tooth loss and risk of end-stage renal disease: a nationwide cohort study. J Periodontol.

[12] Elani HW, Harper S, Thomson WM (2017). Social inequalities in tooth loss: a multinational comparison. Community Dent Oral Epidemiol.

[13] Peres MA, Luzzi L, Peres KG, Sabbah W, Antunes JL, Do LG (2015). Income-related inequalities in inadequate dentition over time in Australia, Brazil and USA adults. Community Dent Oral Epidemiol.

[14] Lee H, Kim D, Jung A, Chae W. Ethnicity, social, and clinical risk factors to tooth loss among older adults in the U.S., NHANES 2011–2018. Int J Environ Res Public Health. 2022;19(4). 10.3390/ijerph19042382 PMC8875070 35206567

[15] Mai X, Wactawski-Wende J, Hovey KM (2013). Associations between smoking and tooth loss according to the reason for tooth loss: the Buffalo OsteoPerio Study. J Am Dent Assoc.

[16] Wiener RC, Shen C, Findley PA, Sambamoorthi U, Tan X (2017). The association between diabetes mellitus, sugar-sweetened beverages, and tooth loss in adults: Evidence from 18 states. J Am Dent Assoc.

[17] Shigli K, Hebbal M, Angadi GS (2009). Relative contribution of caries and periodontal disease in adult tooth loss among patients reporting to the Institute of Dental Sciences, Belgaum, India. Gerodontology.

[18] Guo J, Ban JH, Li G (2018). Status of tooth loss and denture restoration in chinese adult population: findings from the 4th National Oral Health Survey. Chin J Dent Res.

[19] Guangdong Bureau of Statistics. The Seventh National Census of Guangdong Province. 2021. http://stats.gd.gov.cn/dqcrkpc/index.html. Accessed 26 Nov 2022.

[20] Huang L, Wang Z, Wang H (2021). Nutrition transition and related health challenges over decades in China. Eur J Clin Nutr.

[21] Cheng ML, Wang CX, Wang X (2020). Dental expenditure, progressivity and horizontal inequality in Chinese adults: based on the 4th National Oral Health Epidemiology Survey. BMC Oral Health.

[22] Steele JG, Treasure ET, O'Sullivan I, Morris J, Murray JJ (2012). Adult Dental Health Survey 2009: transformations in British oral health 1968–2009. Br Dent J.

[23] Jordan AR, Stark H, Nitschke I, Micheelis W, Schwendicke F. Epidemiological trends, predictive factors, and projection of tooth loss in Germany 1997–2030: part I. missing teeth in adults and seniors. Clin Oral Investig. 2021;25(1):67–76. 10.1007/s00784-020-03266-9 PMC7785540 33219875

[24] Peres MA, Barbato PR, Reis SC, Freitas CH, Antunes JL (2013). Tooth loss in Brazil: analysis of the 2010 Brazilian Oral Health Survey. Rev Saude Publica.

[25] Susin C, Oppermann RV, Haugejorden O, Albandar JM (2005). Tooth loss and associated risk indicators in an adult urban population from south Brazil. Acta Odontol Scand.

[26] Silva-Junior MF, Batista MJ, de Sousa MDLR (2017). Incidence of tooth loss in adults: a 4-year population-based prospective cohort study. Int J Dent.

[27] Panasiuk L, Kosiniak-Kamysz W, Horoch A, Paprzycki P, Karwat D (2013). Tooth loss among adult rural and urban inhabitants of the Lublin Region. Ann Agric Environ Med.

[28] Roberto LL, Silveira MF, de Paula A, Ferreira EFE, Martins A, Haikal DS (2020). Contextual and individual determinants of tooth loss in adults: a multilevel study. BMC Oral Health.

[29] Khazaei S, Keshteli AH, Feizi A, Savabi O, Adibi P (2013). Epidemiology and risk factors of tooth loss among Iranian adults: findings from a large community-based study. Biomed Res Int.

[30] Nguyen TC, Witter DJ, Bronkhorst EM, Truong NB, Creugers NH (2010). Oral health status of adults in Southern Vietnam: a cross-sectional epidemiological study. BMC Oral Health.

[31] Bouma J, van de Poel F, Schaub RM, Uitenbroek D (1986). Differences in total tooth extraction between an urban and a rural area in the Netherlands. Commun Dent Oral Epidemiol.

[32] Andrade FB, Antunes JLF (2018). Trends in socioeconomic inequalities in the prevalence of functional dentition among older people in Brazil. Cad Saude Publica.

[33] Li KY, Okunseri CE, McGrath C, Wong MCM (2018). Trends in self-reported oral health of US adults: National Health and Nutrition Examination Survey 1999–2014. Commun Dent Oral Epidemiol.

[34] Cunha-Cruz J, Hujoel PP, Nadanovsky P (2007). Secular trends in socio-economic disparities in edentulism: USA, 1972–2001. J Dent Res.

[35] Ferreira RC, Senna M, Rodrigues LG, Campos FL, Martins A, Kawachi I (2020). Education and income-based inequality in tooth loss among Brazilian adults: does the place you live make a difference?. BMC Oral Health.

[36] Fukuhara S, Asai K, Kakeno A (2021). Association of education and depressive symptoms with tooth loss. J Dent Res.

[37] Silva Junior MF, Batista MJ, de Sousa MDLR (2019). Risk factors for tooth loss in adults: a population-based prospective cohort study. PLoS ONE.

[38] Bignozzi I, Crea A, Capri D, Littarru C, Lajolo C, Tatakis DN (2014). Root caries: a periodontal perspective. J Periodontal Res.

[39] López R, Smith PC, Göstemeyer G, Schwendicke F (2017). Ageing, dental caries and periodontal diseases. J Clin Periodontol.

[40] AlQobaly L, Sabbah W (2020). The association between periodontal disease and root/coronal caries. Int J Dent Hyg.

[41] Durand R, Roufegarinejad A, Chandad F (2019). Dental caries are positively associated with periodontal disease severity. Clin Oral Investig.

[42] Iwano Y, Sugano N, Matsumoto K (2010). Salivary microbial levels in relation to periodontal status and caries development. J Periodontal Res.

[43] Kinane DF, Jenkins WM, Adonogianaki E, Murray GD (1991). Cross-sectional assessment of caries and periodontitis risk within the same subjectccc. Community Dent Oral Epidemiol.

[44] Yu LX, Wang X, Feng XP (2021). The relationship between different types of caries and periodontal disease severity in middle-aged and elderly people: findings from the 4th National Oral Health Survey of China. BMC Oral Health.

